# HMGB1 release triggered by the interaction of live retinal cells and uveitogenic T cells is Fas/FasL activation-dependent

**DOI:** 10.1186/s12974-015-0389-2

**Published:** 2015-09-22

**Authors:** Guomin Jiang, Yunsong Wang, Juan Yun, Amir Reza Hajrasouliha, Yuan Zhao, Deming Sun, Henry J Kaplan, Hui Shao

**Affiliations:** Department of Ophthalmology and Visual Sciences, Kentucky Lions Eye Center, University of Louisville, 301 E. Muhammad Ali Blvd., Louisville, KY 40202 USA; Department of Ophthalmology, Tangshan Gongren Hospital, Tangshan, Hebei 063000 China; Department of Pharmaceutical Sciences, Sullivan University College of Pharmacy, Louisville, KY 40205 USA; Doheny Eye Institute, Los Angeles, CA 90033 USA

**Keywords:** Autoimmune disease, Autoreactive T cells, Damage-associated molecular patterns, Fas, HMGB1, Immunoregulation, Uveitis

## Abstract

**Background:**

It is not clear how invading autoreactive T cells initiate the pathogenic process inside the diseased organ in T cell-mediated organ-specific autoimmune disease. In experimental autoimmune uveitis (EAU) induced by adoptive transfer of interphotoreceptor retinoid-binding protein (IRBP)-specific T cells in mice, we have previously reported that intraocular inflammation was initiated by infiltrating IRBP-specific T cells that directly interacted with retinal cells and resulted in the active release of high mobility group box 1 (HMGB1), an important member of damage associate molecular patterns (DAMPs). Furthermore, blockade of HMGB1 in our murine model reduced intraocular inflammation via suppression of IRBP-specific T cell functions. These results have demonstrated that HMGB1 is an early and critical mediator of induction of intraocular inflammation. The present study identified the cell surface molecule that triggers HMGB1 secretion.

**Methods:**

Retinal explants from Fas-deficient (Fas^lpr^) and wild-type (Wt) C57BL/6 (B6) mice were cultured with activated IRBP 1–20 peptide-specific T cells or with a Fas-activating antibody (Jo2), and then the level of HMGB1 in culture supernatants were detected by ELISA. In addition, released HMGB1 was examined in the eye of Fas^lpr^ and Wt mice after IRBP-specific T cell transfer. Uveitis was evaluated in the IRBP-specific T cell transferred Fas^lpr^ mice after recombinant HMGB1 was restored within the eye and in the IRBP-specific T cell transferred Wt mice after they were treated with a Fas antagonist (Met12).

**Results:**

In contrast to retinal explants from Wt mice, those from Fas^lpr^ mice did not release HMGB1 after exposure to IRBP-specific T cells or to Jo2. The release of HMGB1 by Wt retinal explants was suppressed by Met 12. Moreover, after IRBP-specific T cell injection, Fas^lpr^ mice did not release HMGB1 in the eye or develop EAU, but intravitreous injection of HMGB1 resulted in intraocular inflammation. Finally, tEAU in Wt mice was attenuated by local treatment with Met 12. Unlike HMGB1, Fas-induced IL-1 and IL-18 were not essential for tEAU induction.

**Conclusion:**

Our results show that interaction of retinal cells with infiltrating uveitogenic T cells leads to rapid release of HMGB1 via the Fas/FasL inflammatory signaling pathway.

## Background

Uveitis is a common cause of human visual disability and blindness. Although the etiology remains unclear, it is generally believed that a T cell-mediated immune response underlies the pathogenesis, and this is supported by the observation that injection of autoreactive T cells into susceptible, syngeneic rodents induces experimental autoimmune uveitis (tEAU) [1–4]. Moreover, studies in the rodent models of EAU induced by immunization with a well-characterized uveitogenic autoantigen, interphotoreceptor retinoid-binding protein (IRBP), have shown that activation of autoreactive T cells is a key pathogenic event in disease induction, progression, and recurrence [2, 5–7]. While a great deal of information is available about the development and activation of autoimmune T cells in the periphery in EAU, it remains unclear how only a few infiltrating uveitogenic T cells can exert a pathogenic effect inside the eye, an “immune privileged” site, leading to tissue destruction [8, 9].

Danger signals are initiated by pathogen-associated molecular patterns (PAMPs) or damage-associated molecular patterns (DAMPs) [10, 11], the latter being actively secreted from inflammatory or stressed cells or passively released from damaged apoptotic or necrotic cells. Both PAMPs and DAMPs can be recognized by pattern-recognition receptors, including TLRs, NOD-like receptors, retinoic acid-inducible gene-like receptors, and receptors for advanced glycation end products [10, 11]. MyD88 is an intracellular adaptor protein required for signaling by most TLRs [12]. The importance of DAMPs in T cell-mediated uveitis was identified as a result of our observation that in the chronic uveitis mouse model induced by transfer of IRBP-specific T cells (tEAU), a model induced without the use of microbial products and resembling human chronic uveitis, mice lacking MyD88 (MyD88^−/−^) are completely resistant to induction of tEAU [13].

In a search for DAMPs that interact with TLRs/MyD88 in tEAU, we previously showed that HMGB1 is an early and critical mediator of IRBP-specific T cell-induced intraocular inflammation, since HMGB1 antagonists reduce ocular inflammation by suppression of uveitogenic T cell functions, such as IRBP-specific T cell proliferation and cytokine production [13]. HMGB1, one of the most important DAMPs released by cells, binds to TLR2 and 4 then MyD88 is recruited by the TLR adaptor protein Mal [14]. We also found that HMGB1 is actively secreted within the eye within 24 h after IRBP-specific T cell transfer as a consequence of direct cell-cell contact between infiltrating IRBP-specific T cells and viable retinal cells [13]. These results led us to search for surface molecules expressed on IRBP-specific T cells and retinal cells that mediate the secretion of HMGB1.

Fas (CD95) is well studied as a death receptor that induces apoptosis through the traditional caspase pathway, presumably one of the mechanisms of immune privilege that protect the eye from severe intraocular inflammation [15–17]. However, recent studies have shown that activation of the Fas/FasL system can also induce release of pro-inflammatory cytokines by macrophages that are independent of conventional caspase-mediated apoptotic signaling [18–21]. Moreover, it was found that Fas activation induces rapid TLR4/IRAK4-dependent release of HMGB1, which contributes to Fas-mediated pro-inflammatory cytokine production by viable macrophages [22]. In the present study, we explored whether Fas also mediated HMGB1 secretion by viable non-lymphoid retinal cells after interaction with activated IRBP-specific T cells. We examined HMGB1 release in the eye and ocular inflammation after injection of IRBP-specific T cells from immunized wild-type (Wt) B6 mice into Fas-deficient (Fas^lpr^) mice and also determined whether early interruption of Fas signaling could suppress tEAU in Wt mice. Our results revealed the importance of Fas/FasL activation in the active secretion of HMGB1 from living retinal cells and its involvement in the early event of intraocular inflammation triggered by uveitogenic T cells.

## Methods

### Animals and reagents

Both on the B6 background, 8 to 10-week-old female C57BL/6 J (B6) mice and Fas^lpr^ and IL-1 receptor-deficient (IL-1RKO) mice were purchased from the Jackson Laboratory (Bar Harbor, ME, USA) and were housed and maintained in the animal facilities of the University of Louisville. Institutional approval was obtained and institutional guidelines regarding animal experimentation followed.

T cells were cultured in RPMI 1640 medium (Mediatech, Manassas, VA, USA) supplemented with 10 % FCS (Hyclone, Logan, UT, USA), 5 × 10^−5^ M 2-ME, and 100 μg/ml of penicillin/streptomycin. The human IRBP_1–20_ peptide (GPTHLFQPSLVLDMAKVLLD) was synthesized by Sigma-Aldrich (St. Louis, MO, USA). The 12-amino acid peptide, mouse Met 12 (HHIYLGAVNYIY), which is a small molecular weight inhibitor of the Fas [23, 24], and a mutant Met 12 (HHGSDHERNYIY) were synthesized by Genemed Synthesis (San Antonio, TX, USA). Recombinant mouse HMGB1 was purchased from R & D System (Minneapolis, MN, USA). The Fas-activating mAb, Jo2, was obtained from BD Pharmingen (San Jose, CA, USA), and the receptor-interacting serine/threonine-protein kinase 2 (RIP2) inhibitor, SB203580, was obtained from Sigma.

### Induction of tEAU

The method used to induce tEAU has been reported previously [4]. Briefly, B6 mice were immunized with IRBP_1–20_ in adjuvant, and 11 days later, T cells were purified from the draining lymph nodes and spleen by passage through a nylon wool column, then 1 × 10^7^ cells in 2 ml of RPMI 1640 medium were added to each well of a six-well plate (Costar, Corning, NY, USA) and stimulated with 20 μg/ml of IRBP_1–20_ in the presence of 1 × 10^7^ irradiated syngeneic spleen cells as APCs. After 2 days, the activated lymphoblasts were isolated by gradient centrifugation on Lymphoprep (Sigma-Aldrich) and injected i.p. in 0.2 ml of PBS into naive B6 recipients (5 × 10^6^ cells/mouse). Experimental mice were killed on day 15, unless stated otherwise.

### Histology

Inflammation of the eye was confirmed by histopathology. Whole eyes were collected, immersed for 1 h in 4 % phosphate-buffered glutaraldehyde, and transferred to 10 % phosphate-buffered formaldehyde until processed. The fixed and dehydrated tissue was embedded in methacrylate, and 5 μm sections were cut through the pupillary-optic nerve plane and stained with H&E. Presence or absence of disease was evaluated blind by examining six sections cut at different levels for each eye. Severity of EAU was scored on a scale of 0 (no disease) to 4 (maximum disease) in half-point increments based on the presence of inflammatory cell infiltration in the iris, ciliary body, anterior chamber, and retina [4].

### Intravitreous injection of Met 12 or recombinant HMGB1

HMGB1 (1 μg/eye) or PBS was injected once into the vitreous on the same day as T cell transfer (day 0), while Met 12 or mutant Met 12 (1 μg/eye) or PBS was injected twice into the vitreous on days 0 and 7. The volume of these intravitreous injections was 2 μl/eye.

### Isolation of retinal explants and co-culture with activated IRBP_1–20_-specific T cells or Jo2

Eyes were collected from naïve Wt or Fas^lpr^ B6 mice and the neural retina isolated and cultured as a retinal explant as described previously [13]. Retinal explants with the inner membrane facing up were cultured at 37 °C with 5 % CO_2_ for 6 h in a 24-well plate in 500 μl of DMEM/F12 medium (Mediatech Inc, Manassas, VA, USA) and 1 % FCS alone or containing either 1 μg/ml of Jo2 or 5x10^4^ activated T cells prepared from IRBP_1–20_-immunized Wt B6 mice on day 11 post-injection and incubated for 2 days with irradiated splenic APCs and IRBP_1–20_ (10 μg/ml), then HMGB1 levels in the supernatant were measured by ELISA, as described below.

### Cell necrosis assay

To ensure that measurement of HMGB1 secretion was not confounded by nonspecific protein release due to cell damage, cytotoxicity was quantified using a standard measurement of lactate dehydrogenase (Sigma LDH kit).

### Intraocular fluid collection

The removed eyeball was immersed in 200 μl of PBS and cut in two, then the cornea, sclera, and lens were discarded, and the rest of the tissue was cut into fine pieces. The suspension containing the aqueous humor, vitreous fluid, and fine pieces of tissue was centrifuged at 500 g for 5 min at 4 °C, and the supernatant immediately was stored in a −80 °C freezer until use. Duplicate samples of about 100 μl from each eyeball were used to measure HMGB1 levels by ELISA as described below [13].

### Measurement of released HMGB1 by ELISA

Protein concentrations of HMGB1 in culture supernatants of retinal explants or intraocular fluids were determined by ELISA kit following the manufacturer’s protocol (USCN Life Science Inc, Missouri City, TX, USA).

### Flow cytometry

For staining of FasL on enriched T cells prepared from the spleens of naive or day 11 post IRBP-immunized B6 mice [4], aliquots of 1 × 10^6^ cells were incubated for 30 min at 4 °C with FITC-conjugated anti-mouse CD3 Ab and phycoerythrin (PE)-conjugated anti-mouse CD178 Ab (eBioscience) or isotype control Ab. Data collection and analysis were performed on a FACSCalibur flow cytometer (BD Biosciences, San Jose, CA, USA) using CellQuest software (BD Biosciences).

### Statistical analysis

Experiments were repeated at least three times. Statistical analysis was performed using an unpaired Student’s *t* test for two sets of data, one-way or two-way ANOVA for three or more means or the Mann-Whitney *U* test for the pathological score of uveitis. A *P* value <0.05 was considered significant. Values determined to be significantly different from those for controls are indicated with asterisks in the figures (*p < 0.05; **p < 0.01).

## Results

### Fas agonist or IRBP-specific T cells promote HMGB1 release from viable retinal cells of Wt, but not Fas^lpr^ mice

**Fig. 1:**
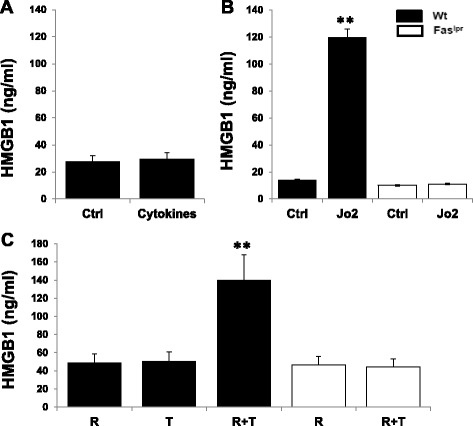
Fas agonist or IRBP-specific T cells promoted HMGB1 release from viable retinal cells of Wt, but not Fas^lpr^ mice. **a** Retina explants collected from Wt B6 mice were cultured with culture medium alone (Ctrl) or containing a cytokine mixture (IFN-γ, IL-17, TNF-α, IL-1β, and IL-6). **b** Retinal explants collected from Wt B6 and Fas^lpr^ mice were cultured with medium alone or containing Jo2. **c** Retinal explants (R) collected from Wt B6 and Fas^lpr^ mice or activated IRBP_1–20_-specific T cells from Wt B6 (T) were cultured alone or in combination (R + T). After 6 h culture of a–c, supernatants were assayed for HMGB1 by ELISA. **p < 0.01 compared to cells cultured with medium alone by one-way ANOVA. The results are representative of those obtained in three separate experiments. In each experiment, each control or experimental group has ≥3 individual wells with 1 retinal explant/well from different mice

Having previously reported that HMGB1 release from viable retinal cells requires direct contact with activated IRBP-specific T cells, we examined which molecules expressed on the retinal cell surface mediate HMGB1 release. We focused on Fas, since it is expressed on the retina [25, 26] and regulates HMGB1 release from viable macrophages [22]. To determine whether Fas activation triggered HMGB1 release from non-lymphoid retinal cells, we prepared retinal explants from Wt and Fas^lpr^ mice and treated them for 6 h with 1 ml of medium (control), an inflammatory cytokine cocktail (500 units of IFN-γ, 100 ng of IL-17, 50 ng of TNF-α, 10 ng of IL-1β, and 100 ng of IL-6), 1 μg/ml of Jo2 (a Fas-activating Ab), or 5x10^4^ activated IRBP_1–20_-specific T cells from immunized Wt B6 mice; the cytokine concentrations used were those recommended by the manufacturer (R & D System). As shown in Fig. 1a, the cytokine mixture did not induce Wt retinal explants to release more HMGB1 than medium alone, consistent with our previous observation that HMGB1 release from retinal cells requires direct contact with activated IRBP-specific T cells [13]. However, Jo2 (Fig. 1b) or activated IRBP-specific T cells (Fig. 1c) significantly increased HMGB1 release by Wt retinal explants, but not Fas^lpr^ retinal explants, indicating that the Fas/FasL interaction or Fas activation regulates HMGB1 release.

**Fig. 2:**
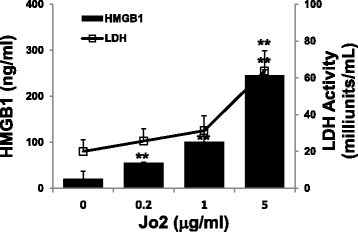
Jo2 does not affect the viability of primary murine retinal cells. Wt retinal explants were incubated for 6 h with medium alone or containing increasing doses of Jo2, then HMGB1 and LDH levels in the supernatants were measured. Values are means ± SEM of three independent experiments. **p < 0.01 compared to retinal explants cultured with medium alone by one-way ANOVA

To check that Jo2 at 1 μg/ml triggered HMGB1 release without cell death, we measured LDH and HMGB1 in culture supernatants of retinal explants exposed to increasing doses of Jo2 (from 0 to 5 μg/ml) for 6 h. As shown in Fig. 2, Jo2 starting at 0.2 μg/ml triggered significant release of HMGB1 from retinal explants, whereas LDH was detected significantly high only when the retinal explant was exposed to 5 μg/ml of Jo2. These results indicate that the concentration of Jo2 used in this study induced Fas-mediated HMGB1 release from retinal cells via a mechanism that did not involve, or depend on, cell death.

### Transfer of IRBP-specific T cells into Fas^lpr^ mice does not lead to HMGB1 release in the eye or development of tEAU

**Fig. 3:**
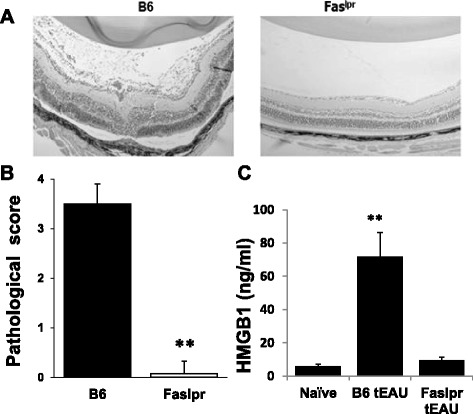
Fas^lpr^ mice do not develop ocular inflammation or release HMGB1 after IRBP-specific T cell transfer. Wt and Fas^lpr^ mice were injected with IRBP_1–20_-specific T cells and examined on day 15 (**a**–**c**) post-injection. **a** Representative ocular histopathology with H & E staining of Wt and Fas^lpr^ eye sections, original magnification ×100. **b** Pathological score for the two groups (*n* = 12 mice) presented as the mean ± SEM. **p < 0.01 compared to Wt mice using the Mann-Whitney *U* test. **c** HMGB1 levels in the intraocular fluid (6 eye/group) measured by ELISA. **p < 0.01 compared to naïve mice by one-way ANOVA

To demonstrate that Fas-induced HMGB1 release is important for tEAU induction, we injected activated IRBP-specific T cells from immunized Wt B6 mice into Wt and Fas^lpr^ mice and examined intraocular inflammation and HMGB1 at day 15. In contrast to Wt mice, Fas^lps^ mice exhibited no, or only very mild, ocular inflammation (Fig. 3a, b) and only very low levels of HMGB1 in the ocular fluid (Fig. 3c).

### Blockade of Fas signaling using Met 12 reduces HMGB1 release and attenuates tEAU in Wt mice

**Fig. 4:**
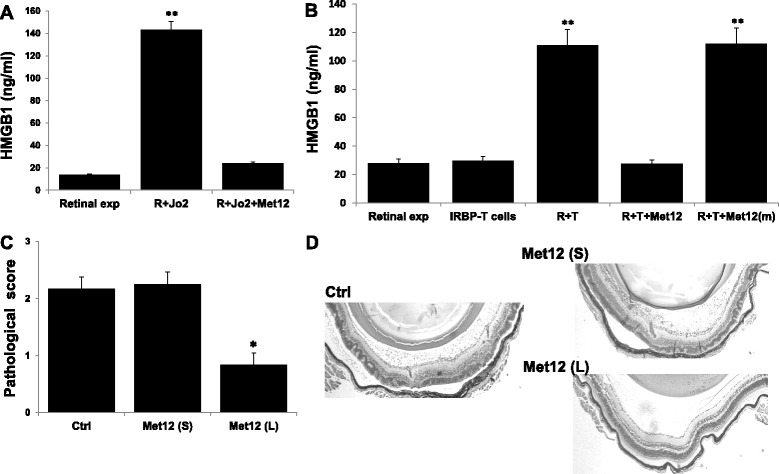
Blockade of Fas signaling on retinal cells in Wt mice inhibits HMGB1 release and tEAU induction. **a**, **b** Retinal explants (or R) from Wt mice were cultured for 6 h with or without Jo2 (**a**) or activated IRBP_1–20_-specific T cells (**b**) in the presence or absence of 1 μg/ml of Met 12 (**a**, **b**) or the mutant Met 12 (m) (**b**), then HMGB1 levels in the culture supernatant were measured by ELISA. **p < 0.01 compared to retinal explants treated with medium by one-way ANOVA. **c**, **d** Wt mice injected with IRBP_1–20_-specific T cells were injected systemically (S) or intravitreously (L) with Met 12 or PBS (Ctrl) and examined on day 15; **c** shows the pathological score for each group (*n* = 12 mice) presented as the mean ± SEM, *p < 0.05 compared to PBS treatment using the Mann-Whitney *U* test, while **d** shows representative ocular histopathology after H & E staining, original magnification, ×100

To further confirm that Fas is important for cell-cell contact-induced HMGB1 release and to explore possible therapy for inhibition of HMGB1 release by blocking Fas activation, we used a novel inhibitor of the Fas pathway, Met 12, a 12-amino acid peptide containing the YLGA motif (HHIYLGAVNYIY) in the N-terminal region of the extracellular domain of the α chain of Met that binds to Fas and prevents its activation by sequestering it [23, 24]. A mutant peptide, HHGSDHERNYIY, that cannot bind to Fas was used as a control. As shown in Fig. 4, Met 12 suppressed HMGB1 release from Wt retinal explants cultured with Jo2 (Fig. 4a), and Met 12, but not the mutant peptide, suppressed HMGB1 release from Wt retinal explants cultured with activated IRBP-specific T cells (Fig. 4b). Importantly, severity of tEAU was reduced by intravitreous, but not systemic (intraperitoneal), injection of Met 12 on days 0 and 7 after IRBP-specific T cell transfer (Fig. 4c, d).

### Local administration of HMGB1 restores development of severe tEAU in Fas^lpr^ mice

**Fig. 5:**
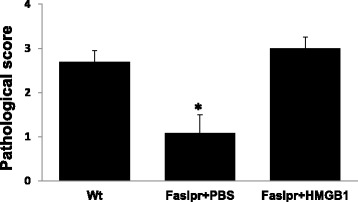
Intravitreous injection of HMGB1 allows induction of tEAU in Fas^lpr^ mice. Fas^lpr^ mice injected with IRBP_1–20_-specific T cells were intravitreously injected with HMGB1 (1 μg/eye) or PBS (*n* = 9) on the day of cell transfer and examined on day 15; Wt mice (*n* = 9) injected with IRBP_1–20_-specific T cells were used as the positive control. The pathological score for each group is presented as the mean ± SEM. *<*p* < 0.05 compared to Wt mice injected with IRBP_1–20_-specific T cells and injected intravitreously with PBS using the Mann-Whitney *U* test

To determine whether very mild ocular inflammation seen in Fas^lpr^ mice (Fig. 3) following cell transfer was a result of low extracellular HMGB1 levels, we injected HMGB1 or PBS into the vitreous of Fas^lpr^ mice on the same day as the transfer of activated IRBP_1–20_-specific T cells and found that injection of HMGB1, not PBS, resulted in similar levels of intraocular inflammation to those in Wt mice injected with IRBP_1–20_-specific T cells (Fig. 5).

### A RIP2 inhibitor reduces Fas-induced HMGB1 release by living retinal cells

**Fig. 6:**
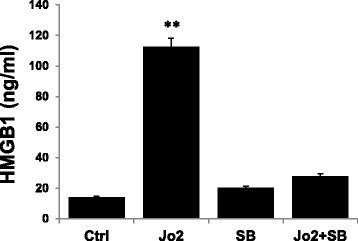
An RIP2 inhibitor reduces Jo2-induced HMGB1 release by Wt retinal cells. Retinal explants from Wt mice were cultured for 6 h with medium or medium containing 1 μg/ml of Jo2 in the presence or absence of an RIP2 inhibitor (SB) (1 μg/ml), then HMGB1 levels in the culture supernatants were measured by ELISA. **p < 0.01 compared to retinal explants treated with medium by one-way ANOVA

RIP2 is a receptor-interacting serine/threonine kinase with a C-terminal caspase activation and recruitment domain (CARD), which contains a highly conserved tyrosine phosphorylation site, phosphorylation of which plays a critical role in Fas-mediated apoptosis [27]. Since RIP2 can also induce activation of NF-kB, thus modulating the inflammatory function of epithelial cells [28], we examined whether RIP2 regulated Fas-mediated HMGB1 release from live retinal cells and thus promoted ocular inflammation by treating Wt retinal explants with Jo2 in the presence or absence of the RIP2 inhibitor SB203580 and measured HMGB1 levels in the culture supernatants. As shown in Fig. 6, SB203580 significantly inhibited Jo2-induced HMGB1 release from retinal explants; similar results were observed using retinal astrocytes treated with Jo2 with or without SB203580 (data not shown).

### Fas-induced IL-1β release is not required for development of IRBP-specific T cell-induced intraocular inflammation

**Fig. 7:**
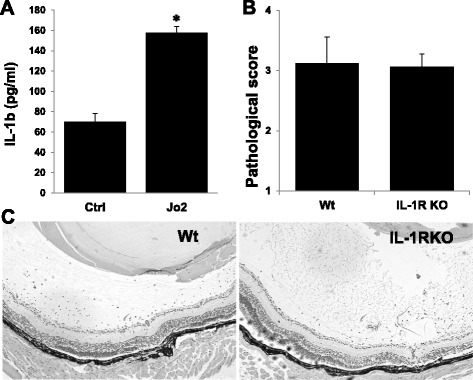
Fas-induced IL-1β release is not needed for tEAU induction. **a** Retinal explants collected from Wt mice were cultured for 6 h with medium (Ctrl) or medium containing 1 μg/ml of Jo2, then the culture supernatants were assayed for IL-1β by ELISA. *p < 0.05 compared to retinal explants treated with medium alone by one-way ANOVA. **b**, **c** Wt and IL-1RKO mice were injected with IRBP_1–20_-specific T cells and examined on day 21. **b** shows the pathological score for each group (*n* = 9 mice) presented as the mean ± SEM and **c** shows representative ocular histopathology with H & E staining, original magnification, ×100

Fas activation promotes release of inflammatory cytokines, such as IL-1β and IL-18 [29], both of which recruit MyD88, an adaptor protein linking to downstream signaling pathways. When Wt retinal explants were incubated with or without Jo2 for 6 h, HMGB1 (Fig. 1) and IL-1β (Fig. 7a) were detected in the culture supernatants. Neutralizing anti-HMGB1 antibody is known to reduce the severity of tEAU in Wt B6 mice, showing that HMGB1 is involved in this process [13]; however, in the present study, after injection of IRBP_1–20_-specific T cells in IL-1RKO and IL-18KO mice, IL-1RKO developed similar severe ocular inflammation as Wt mice (Fig. 7b, c) so did IL-18KO mice (data not shown), indicating that IL-1β and IL-18 were not involved in the effector phase of intraocular inflammation.

## Discussion

The Fas/FasL system has been mainly studied for its role in caspase-dependent, apoptotic programmed cell death [30] to maintain tissue/cell homeostasis. Previous studies on the eye have shown that both FasL and Fas are expressed on ocular tissues and contribute to the immunologically privileged status of the eye by causing apoptosis of invading leukocytes and protects the eye from immune-mediated damage [15–17, 26] or play a role in tissue damage by promoting apoptotic death of ocular cells.[17, 31, 32]. However, the Fas/FasL system is being increasingly recognized for its ability to trigger inflammation [29]. Numerous studies have demonstrated that activation of Fas signaling in a variety of non-lymphoid cells, including colonic and lung epithelial cells [33, 34], hepatocytes [35], synoviocytes [36], macrophages [20], and fibroblasts [37], can lead to the expression and release of inflammatory factors in vitro and in vivo, in particular, at the early stage of inflammation development. Such factors may, in turn, recruit inflammatory cells, exacerbating the inflammatory process. Accordingly, Fas/FasL-mediated inflammation has been shown to play an important role in the pathogenesis of several diseases, including acute respiratory distress syndrome [34], cystic fibrosis [38], arthritis [36, 39], and cancer [40], all of which have an underlying inflammatory component. Moreover, many chronic inflammatory diseases are attenuated in mice lacking Fas or FasL (*gld*) [39, 41, 42]. For example, compared to Wt B6 mice, *gld* and *lpr* mice are highly resistant to the development of experimental autoimmune encephalomyelitis (EAE) [43–45] and EAU [46], which share essential cellular mechanisms, indicating involvement of Fas/FasL in the T cell-mediated tissue inflammation.

Our results clearly demonstrate that Fas is required for active and rapid release of HMGB1 from tissue retinal cells via cell-cell interaction with activated uveitogenic T cells. Released HMGB1 either alone or in combination with other pro-inflammatory mediators triggers inflammatory cascades in the eye, probably by enhancing and sustaining the pathogenicity of IRBP_1–20_-specific T cells. The in vitro results that Fas on retinal cells mediates HMGB1 release was further supported by our in vivo studies using Fas knockout mice, in which transfer of IRBP_1–20_-specific T cells failed to trigger HMGB1 release or induce tEAU, while local HMGB1 injection restored susceptibility to induction of ocular inflammation. These data are the first to implicate HMGB1 in Fas-mediated HMGB1 release from tissue cells in response to infiltrating autoreactive T cells and provide a possible explanation for the observation that Fas^lpr^ mice do not develop EAU after immunization with IRBP antigen or adoptive transfer of IRBP-specific T cells [46] (Fig. 3). The wild expression of Fas on photoreceptor cells, retinal pigment epithelium [26], microglia [47], and astroglia [48] of the retina correlated with the expression pattern of HMGB1 [13]. Our study suggested that one of the mechanisms that a few T cells initiate autoimmune uveitis is that IRBP-specific T cells interact with parenchymal cells such as residential DCs [49], microglia [50], astrocytes [51], and Müller cells [52], resulting in the subsequent production of HMGB1 by those cells, mediated by Fas/FasL, an early event in the pathogenesis of intraocular inflammation. Most adoptively transferred disease-inducing T cells require “licensing for pathogenicity” in the lung and other organs in order to induce disease in target organs [53, 54]—“a hub-and-spoke pattern” [55]. Both processes of “licensing for pathogenicity” and of HMGB1 production could take place during the induction of effector phase of EAU. The release of intraocular HMGB1 on day 1 may attract the migration of “licensed” pathogenic T cells to the eye, and the later increase of HMGB1 could be related to the involvement of these T cells and the damage they invoke in the retina.

The form of HMGB1 should be assessed as this has a major effect on promotion of either pro-inflammatory cytokine production or chemo-attraction of inflammatory cells, although neutralization of HMGB1 inhibited tEAU. Our results complement our current understanding of the Fas/FasL system in the eye, i.e., that it is not involved only in induction of apoptosis of infiltrating leukocytes or tissue cells, but also in initiation of inflammation, in particular, during the early stage of disease development in the eye. Different initial molecular events might lead to the activation of different molecular mechanisms resulting in the transmission of either apoptotic or non-apoptotic signals. The molecular process by which Fas is switched from an inflammatory role to an apoptotic function is not known, and the paradoxical roles of the Fas/FasL interaction in stimulating apoptosis of invading leukocytes in the eye and in promoting HMGB1 release and inflammation in the eye need to be explored.

How the Fas signaling pathway triggers HMGB1 release is not known. Met 12, a 12-amino acid peptide containing the YLGA motif in the N-terminal region of the extracellular domain of the α chain of Met, a tyrosine kinase receptor for hepatocyte growth receptor, is a small molecular weight inhibitor of Fas [23, 24] and acts as an inhibitor of the apoptosis-activating Fas/FasL pathway in a transformed photoreceptor cell line (661 W cells) and in an animal model of retinal detachment [23]. We tested the inhibitory effect of Met 12 on Fas-mediated HMGB1 release, and the subsequent intraocular inflammation and the results, shown in Figs. 4 and 5, support our hypothesis that Fas on retinal cells mediates HMGB1 release, which can be blocked by Met 12. Our results showed that adoptive transfer of IRBP-specific T cells reduced EAU severity in the Fas^lpr^ mice compared with WT mice (Fig. 3), whereas blocking of Fas systemically using Met 12 did not have a similar effect on EAU disease compared with controls (Fig. 4c). It has been repeatedly observed that experimental results using KO mice are frequently different when compared to blocking agents (or antibodies). In Fas KO mice, there is both systemic and intraocular deletion of Fas so that infiltrating T cells cannot interact with Fas on parenchymal ocular cells, resulting in inhibition of EAU. In contrast, the dose of Met used systemically may be too low to block Fas expressed within the eye, and/or Met administered systemically may be degraded before entering the eye. Support for these explanations is provided by the observation that the intraocular injection of Met does block Fas/FasL interaction between retinal cells and infiltrating T cells.

Another new finding was that RIP2, a receptor-interacting serine/threonine kinase with a C-terminal caspase activation and recruitment domain (CARD) that plays a critical role in Fas-mediated apoptosis [27], is also involved in Fas-mediated HMGB1 release (Fig. 6). Our finding that downstream molecules of the Fas signaling pathways, such as Met and Rip2, regulate both apoptosis and inflammation complicates the use of pharmaceutical agonists or antagonists for Fas-mediated pathological events. Further studies on the role of Fas/FasL in inflammation and apoptosis in immune responses should result in improved immunotherapies based in Fas/FasL and their signaling molecules.

Although the Fas activator Jo2 triggered release of both HMGB1 and IL-1 from retinal cells (Figs. 1 and 7), we have previously shown that HMGB1 antagonists reduce intraocular inflammation induced by injection of IRBP_1–20_-specific T cells [13], whereas, transfer of IRBP_1–20_-specific T cells into IL-1RKO mice induced disease in all six mice (Fig. 7). Together, these results show that HMGB1, but not IL-1, is required for intraocular inflammation triggered by infiltrating effector autoreactive T cells. In contrast, IL-1RKO mice are resistant to active induction of EAU by immunization with IRBP_1–20_ and complete Freund’s adjuvant (data not shown and [56, 57]. These results suggest that the IL-1R is required for the generation of pathogenic T cells, in particular Th17 cells [58, 59] but is not needed for the subsequent pathogenic events occurred in the eye, i.e., in the effector phase of EAU. Similarly, in an EAE model mice used to study multiple sclerosis in humans, the IL-1R was found to be required for induction of EAE by active immunization with the antigen MOG but not for induction of EAE induced by transfer of MOG-specific T cells [60]. Together, these results suggest that, once disease induced by pathogenic Th17 cells has been established, reducing IL-1 levels may not be an effective means of treatment, whereas blockade of HMGB1 and its related signaling molecules might achieve the therapeutic goal.

## Conclusion

These data demonstrate an early event in the pathogenesis of intraocular inflammation initiated by a few infiltrating uveitogenic T effector cells, that is, uveitogenic T cells interact with residential APCs, leading to the subsequent production of HMGB1 by those cells via the Fas/FasL inflammatory signaling pathway. Blockade of HMGB1 and its related signaling molecules might achieve the therapeutic goal in T cell-mediated intraocular inflammation.

## Abbreviations

DAMPs: damage-associated molecular patterns
EAU: experimental autoimmune uveitis
Fas^lpr^: Fas-deficient mice
HMGB1: high mobility group box 1
IRBP: interphotoreceptor retinoid-binding protein
MyD88: myeloid differentiation factor 88
PAMPs: pathogen-associated molecular patterns
Rip2: receptor-interacting serine/threonine-protein kinase 2
tEAU: activated IRBP-specific T cell-induced EAU
TLRs: Toll-like receptors
